# An association study of *SERPING1* gene and age-related macular degeneration in a Han Chinese population

**Published:** 2010-01-10

**Authors:** Fang Lu, Peiquan Zhao, Yinchuan Fan, Shibo Tang, Jianbin Hu, Xiaoqi Liu, Xian Yang, Yiye Chen, Tao Li, Chuntao Lei, Jiyun Yang, Ying Lin, Shi Ma, Chunyong Li, Yi Shi, Zhenglin Yang

**Affiliations:** 1 Center for Human Molecular Biology & Genetics, Sichuan Academy of Medical Sciences & Sichuan Provincial People’s Hospital, Sichuan, China; 2 Department of Ophthalmology, Xinhua Hospital, Shanghai Jiao Tong University, Shanghai, China; 3 Department of Ophthalmology, Sichuan Academy of Medical Sciences & Sichuan Provincial People’s Hospital, Sichuan, China; 4 Zhongshan Ophthalmic Center, Guangdong, China; 5 Department of Ophthalmology, School of Medicine, Qingdao University, Qingdao, China

## Abstract

**Purpose:**

Single nucleotide polymorphisms (SNPs) in the complement component 1 inhibitor (*SERPING1*) gene have been shown to be significantly associated with age-related macular degeneration (AMD) in Caucasian populations. A replication study of an association between these SNPs and AMD in a Chinese population is reported in this study.

**Methods:**

Six SNPs, including rs2511990, rs1005510, rs11546660, rs2511989, rs2511988, and rs4926 in *SERPING1* were genotyped in a Han Chinese subject group using the SNaPshot method of ABI. This subject group was composed of 194 patients with choroidal neovascularization (CNV or wet) AMD, 78 patients with soft drusen, and 285 matched controls. P values of the SNPs were calculated using an additive model. Haplotype frequencies between cases and controls were compared by χ2 analysis. The haplotype analysis was performed using Haploview 4.0.

**Results:**

None of the six SNPs showed significant association with AMD. None of the major haplotypes were observed to be significantly associated with AMD or choroidal neovascularization AMD (CNV) after a stringent Bonferroni correction.

**Conclusions:**

We demonstrate that SNPs in *SERPING1* are not significantly associated with AMD in the mainland Han Chinese population.

## Introduction

Age-related macular degeneration (AMD) is a leading cause of blindness in the elderly population, characterized as chronic and progressive degeneration of photoreceptors, the underlying retinal pigment epithelium (RPE), Bruch’s membrane, and possibly, the choriocapillaris in the macula [1,2]. AMD is divided clinically into dry and wet AMD. Patients with dry AMD present with cellular debris (drusen) in or under the retinal pigment epithelium (RPE), irregularities in the pigmentation of the RPE, or geographic atrophy (GA). Patients with exudative or wet AMD are characterized by serous detachment of the RPE or choroidal neovascularization (CNV), or both [1,2]. Advanced AMD, including geographic atrophy or exudative disease, can cause severe vision loss.

It is believed that AMD is a complex disorder caused by the interaction of multiple genetic and environmental risk factors [3–7]. Identification of AMD related genes has been tremendously successful. Complement pathway genes, including complement factor *H* (*CFH*) [2,8–13], *C2/CFB* [14–16], and *C3* [15–17], have been confirmed by many replication studies. The *LOC387715/HTRA1* gene has also been verified as a major AMD locus in different populations [18–24]. Recently, SNPs in the serpin peptidase inhibitor, clade G (C1 inhibitor) member 1 (*SERPING1*) gene showed highly significant genotypic association with age-related macular degeneration in two Caucasian populations [25]. Unfortunately, this finding could not be replicated by other studies [26–34].

To further analyze the association of *SERPING1* and AMD, we investigated the association between SNPs in this gene and AMD in a mainland Han Chinese population.

## Methods

### Subjects

The Institutional Review Boards of the Sichuan Provincial People’s Hospital, Xinhua Hospital of Shanghai Jiao Tong University, and Zhongshan Ophthalmic Center, China approved this study. All subjects provided informed consent before participation in the study. AMD patients and normal age-matched controls, including individuals with a normal eye examination (individuals age 60 years or older with no drusen or RPE changes), were recruited in the ophthalmology clinic at Sichuan Provincial People’s Hospital, Xinhua Hospital of Shanghai Jiao Tong University, and Zhongshan Ophthalmic Center, China. All participants went through a standard examination protocol as in the previous description [19,24,27]. Grading was performed using a standard grid classification suggested by the International ARM Epidemiological Study Group for age-related maculopathy (ARM) and the age-related macular degeneration group [27]. All abnormalities in the macula were characterized according to the type, size and number of drusen, and hyperpigmentation or hypopigmentation, as well as AMD stages as defined by AREDS 1–5 stages. Patients with clinical features of AMD and CNV (CNV from other causes was excluded), with or without drusen, were diagnosed as wet AMD patients. Patients with only soft drusen were diagnosed as drusen (dry AMD) patients. In total, 194 wet AMD patients (Eight from Zhongshan Ophthalmic Center, 28 from Xinhua Hospital, 158 from of the Sichuan Provincial People’s Hospital), 78 soft drusen patients (all from of the Sichuan Provincial People’s Hospital), and 285 normal matched controls (Eight from Zhongshan Ophthalmic Center, 30 from Xinhua Hospital and 247 from of the Sichuan Provincial People’s Hospital) were recruited. In the normal matched controls, all individuals underwent an eye exam, no signs of early AMD, such as soft drusen or irregular pigmentations of the RPE in the macular area, were observed. Clinical information about the cases and controls is listed in Table 1.

**Table 1:** Characteristics of amd cases and controls matched for ages and ethnicity.

**Subject**	**Total number**	**Male**	**Female**	**Average age**
All AMD (CNV+drusen)	272	126	146	68.2±9.8
CNV	194	90	104	69.4±12.2
drusen	78	36	42	68.7±8.7
Controls	285	132	153	68.4±7.2

### Selection of tag and functional SNPs

We used the data of the Han Chinese Beijing population in HapMap3 of the international HapMap project and previous studies to select tag SNPs or functional SNPs in *SERPING1* [26,28] for this study. Six SNPs were selected for genotyping, including rs2511990 upstream of the transcription start site (−2877 bases), rs1005510 in intron 2, rs11546660 (A56V) in exon 3, rs2511989, rs2511988 in intron 6, and rs4926 (M480V) in exon 8.

### Genotyping

Blood from each subject was drawn and collected in an EDTA-containing tube. Genomic DNA was extracted from the blood by a Gentra Puregene Blood DNA kit (Minneapolis, MN). SNP genotyping was performed by the dye terminator-based SNaPshot method (Applied Biosystems, Foster City, CA). SNP analysis was performed on the ABI 3130 genetic analyzer (Applied Biosystems). Genotypes of the SNPs were determined by Genemapper software (Applied Biosystems). All SNPs reported in this manuscript had a genotyping success rate >96 percent and accuracy as judged by random re-genotyping of 10 percent of the samples in the subject group. Six SNPs in *SERPING1* were genotyped. The PCR and SNaPshot primers are listed in Table 2.

**Table 2:** Genotyping primers.

**SNP**	**PCR Primer Forward**	**Snapshot Primer**
rs2511989	F: TTCACAGCCTACCTTTCC	CCCTGGGTTTAATACAGGGGTTGTCAACTC
	R: CAGCCTCAATCATAATACCA	
rs2511990	F: AAGCTGGAGCTGAAACTG	GTTCTCTTCCCACTGGGAGCAGGTCTAGGATTTCTC
	R: GGAAGAGGGATTCTGTGG	
rs1005510	F: TTCTTACTACGAGGCACA	ATGTGGAAAATGTCCTGTACAAGAGAGTAATTTCTGACAGTGC
	R: TAAATCAAGGAGCACAAG	
rs2244169	F: TGGCGTGAACCCTGGAGA	CAGCCAGAAAAGTTTTACAAAGCACGTATATGAC
	R: AGGTGGGAGGATTGCTTG	
rs2511988	F: AGTGGGCTGGAACTTGGA	ATTGTGGGAGAGCTGCAGCTGCCCCACCTAGAAAATAAGAGATGCA
	R: GCATTGTGACAGAGGGTG	

### Haplotype analysis

Haplotype analysis was performed using Haploview 4.0. We performed the haplotype analysis following the instructions from the Broad Institute. If the genotype was not available, the genotype was set as 0.

### Statistical analysis

Hardy–Weinberg equilibrium (HWE) for each SNP polymorphism was tested by the χ^2^ test with df=1. P values of the SNPs were calculated using an additive model. Haplotype frequencies between cases and controls were compared by χ^2^ analysis. The unadjusted odds ratios of the alleles and genotypes were estimated by the χ^2^ test. All statistical analyses were performed using the software SPSS, (SPSS, Chicago, IL) version 10.0 [18–24].

## Results

### Single nucleotide polymorphism analysis

All six SNPs selected were successfully genotyped and all of these SNPs were within HWE in both case (p>0.001, Table 3) and control groups (p>0.05, Table 3). The SNP frequencies in this study were similar to those of Han Chinese Beijing (HCB) available in HapMap3 in the International HapMap Project. None of the six SNPs showed significant association with AMD or subphenotypes of AMD including wet AMD or soft drusen, which are landmarks of early AMD even before a stringent Bonferroni correction (p≥0.05, Table 3). SNP rs2511989 was reported to be the most significant association of SNP in the *SERPING1* gene with AMD in previous studies [25]. Although rs2511989 showed high polymorphism, no association between this SNP and AMD was observed in the Chinese population (p=0.76 for all AMD; p=0.61 for CNV AMD; p=0.77 for soft drusen).

**Table 3:** Association between subphenotypes of AMD ANS SNPS in *SERPING1* in the Han Chinese subject group.

**SNP (risk allele)**	Physical location (Chr.11)*	**Phenotype**	**Genotype count**			**Allele frequency**	**HWE**	**Trend p-value**
rs2511990 (T)	57119284	CVN+Drusen	CC:211	CT:50	TT:10	0.13	0.003	0.59
		CNV	CC:148	CT:35	TT:10	0.14	0.002	0.3
		Drusen	CC:63	CT:15	TT:0	0.1	0.643	0.47
		Control	CC:226	CT:51	TT:8	0.12	0.068	
rs1005510 (G)	57123798	CVN+Drusen	AA:140	AG:117	GG:15	0.27	0.135	0.44
		CNV	AA:101	AG:81	GG:12	0.27	0.724	0.49
		Drusen	AA:39	AG:36	GG:3	0.27	0.312	0.58
		Control	AA:134	AG:131	GG:16	0.29	0.087	
rs11546660 (C)	57124043	CVN+Drusen	TT:249	CT:21	CC:0	0.04	0.506	0.05
		CNV	TT:178	CT:16	CC:0	0.04	0.836	0.1
		Drusen	TT:71	CT:5	CC:0	0.03	0.957	0.13
		Control	TT:245	CT:35	CC:1	0.07	0.978	
rs2511989 (A)	57134901	CVN+Drusen	GG:198	AG:57	AA:5	0.13	0.706	0.76
		CNV	GG:147	AG:42	AA:5	0.13	0.644	0.61
		Drusen	GG:51	AG:15	AA:0	0.08	0.298	0.77
		Control	GG:215	AG:63	AA:3	0.12	0.791	
rs2511988 (C)	57135746	CVN+Drusen	TT:155	CT:103	CC:14	0.25	0.557	0.99
		CNV	TT:110	CT:73	CC:11	0.24	0.971	0.89
		Drusen	TT:45	CT:30	CC:3	0.23	0.763	0.77
		Control	TT:155	CT:118	CC:9	0.24	0.025	
rs4926 (A)	57138565	CVN+Drusen	GG:201	AG:65	AA:6	0.14	0.784	0.43
		CNV	GG:147	AG:42	AA:5	0.13	0.644	0.7
		Drusen	GG:54	AG:23	AA:1	0.16	0.689	0.25
		Control	GG:209	AG:63	AA:3	0.13	0.766	

### Haplotype association analysis

We then performed haplotype analysis using Haploview 4.0, and 14, 15, and 14 haplotypes were observed in the AMD-control, wet AMD-control, and drusen-control groups, respectively. We found that haplotype TGTGCG and haplotype CGCGCG were shown to have a significant difference between both AMD-control (p=0.0064, p=0.006, respectively, Table 4) and wet AMD-control groups (p=0.0042, p=0.025, respectively, Table 4). The haplotype CGTGCG was shown to have a significant difference between both AMD-control (p=0.0102, Table 4) and drusen-control (p=0.032, Table 4). In addition, the haplotype CGTGTA was shown to have a significant difference between wet AMD and controls (p=0.026, Table 4). But none of the haplotypes were shown to have a significant difference between cases and controls (p>0.05, Table 4) after a stringent Bonferroni correction. On the other hand, the haplotype CGTGCA was shown to be significantly associated with soft drusen in our subject group (p=7.87x10^−5^, Table 4) with frequencies of 0.11 in cases and 0.03 in controls, even after a stringent Bonferroni correction (p=0.0011, Table 4). This haplotype conferred a 3.72-fold (95% CI: 1.83–7.54) increased likelihood of dry AMD (Table 4). Additionally, the haplotype CGTACA was also shown to have a significant difference between both all AMD-control and wet AMD-control groups (p<0.05, Table 4) after a stringent Bonferroni correction. However, the frequency of this haplotype was low and it was absent in the controls.

**Table 4:** *SERPING1* haplotype association with AMD in the Han Chinese subject group.

**Type of AMD**	**Haplotype**	**Frequency**		**Haplotype association (p value)**	**Bufferoni correction**	**Odds ratio (95% CI)**
		**Case**	**Control**	** **	**(p value)**	
All AMD	H1:CATGTG	0.63	0.57	0.0609		
	H2:CGTGTG	0.04	0.06	0.1091		
	H3:TGTGCG	0.06	0.02	0.0064	0.0900	
	H4:TGTACG	0.03	0.04	0.3301		
	H5:CGTGCA	0.04	0.03	0.2641		
	H6:CGTGCG	0.02	0.05	0.0102	0.1428	
	H7:CATGTA	0.02	0.03	0.4105		
	H8:CATATG	0.02	0.03	0.5689		
	H9:TATGTG	0.02	0.01	0.3679		
	H10:CGCGCA	0.02	0.01	0.5900		
	H11:CGTACA	0.03	0.00	0.0008	0.0112	
	H12:CGTACG	0.01	0.02	0.4070		
	H13:CGTGTA	0.01	0.02	0.0628		
	H14:CGCGCG	0.00	0.02	0.0060	0.0840	
Wet AMD	H1:CATGTG	0.63	0.57	0.0717		
	H2:CGTGTG	0.03	0.06	0.0946		
	H3:TGTACG	0.04	0.04	0.7668		
	H4:TGTGCG	0.06	0.02	0.0042	0.0630	
	H5:CGTGCG	0.02	0.05	0.0620		
	H6:CATGTA	0.03	0.03	0.6366		
	H7:CGTGCA	0.02	0.03	0.5447		
	H8:CATATG	0.02	0.03	0.2741		
	H9:CGCGCA	0.02	0.01	0.5848		
	H10:TATGTG	0.02	0.01	0.3616		
	H11:CGTACG	0.01	0.02	0.6028		
	H12:CGCGCG	0.00	0.02	0.0256	0.3840	
	H13:CGTACA	0.03	0.00	0.0003	0.0045	
	H14:CATGCG	0.01	0.01	0.3513		
	H15:CGTGTA	0.00	0.02	0.0263	0.3950	
Drusen AMD	H1:CATGTG	0.62	0.57	0.2702		
	H2:CGTGTG	0.04	0.06	0.5894		
	H3:CGTGCA	0.11	0.03	7.87E-05	1.10E-03	3.72 (1.83–7.54)
	H4:CGTGCG	0.01	0.05	0.0317	0.4438	
	H5:TGTACG	0.01	0.04	0.0909		
	H6:CATATG	0.04	0.03	0.6489		
	H7:TGTGCG	0.05	0.02	0.1275		
	H8:CATGTA	0.02	0.03	0.3929		
	H9:CGTGTA	0.01	0.02	0.6998		
	H10:CGCGCG	0.00	0.02	0.0894		
	H11:TATGTG	0.02	0.01	0.3297		
	H12:CGCGCA	0.01	0.01	0.8976		
	H13:CGTACG	0.01	0.02	0.4630		
	H14:CATGCG	0.00	0.01	0.2656		

## Discussion

Although genes in complement pathways, including *CFH*, *C2/BF*, and *C3* [2,8–17] and chr.10q26 (*LOC387715/HTRA1*) [18–24], have been identified as related to AMD, these loci could not explain all genetic contributions to AMD, suggesting that additional genetic variants related to AMD have not yet been found. Based on the candidate gene approach, Ennis et al. [25] reported that SNPs in *SERPING1* were significantly associated with AMD in two Caucasian populations. Additional evidence for *SERPING1* involving AMD includes: 1) *SERPING1* gene encoding C1INH plays an important role in complement pathways, which have been confirmed to participate in the pathogenesis of AMD; and 2) *SERPING1* was expressed in both retinal and RPE-choroid layers in RT–PCR and immunofluorescence studies [25,29]. AMD affection status was correlated with increased abundance of choroidal C1INH [29]. Complement activation pathways include lectin, classical and alternative pathways. *SERPING1* encodes C1INH, an inhibitor of the classical and lectin pathways of complement activation. The classical complement pathway is initiated by the C1 complex, which comprises a C1q hexamer complex with a zymogenic (C1r)_2_-(C1s) _2_). SERPING1 irreversibly inhibits C1r and C1s, MASP-1 (mannan-binding lectin serine peptidase 1), and MASP-2 (mannan-binding lectin serine peptidase 2, the C1s ortholog in the lectin pathway), as well as modulating the complement activation through inhibition unrelated to proteases [30–33]. However, Park et al. [26] were unable to replicate the association between the genetic variation in *SERPING1* and AMD in two large and well characterized Caucasian subject groups, and Allikmets et al. [34] were also unable to replicate the association between rs2511989 in *SERPING1* and AMD. Additional replication studies, especially of a different ethnicity, are important to determine if *SERPING1* is really associated with AMD. None of the six SNPs showed significant association with AMD and none of the major haplotypes were observed to be significantly associated with AMD or choroid neovascularization AMD (CNV) after a stringent Bonferroni correction in our study, suggesting that *SERPING1* may not be related to AMD in the Han Chinese population. In the haplotype analysis, none of the SNPs tagged the significant haplotypes. Because half of samples’ genotype data for rs11546660 and rs4926 was not available in the HapMap3 for the Chinese, we cannot compare the haplotype frequencies to those in the HapMap. Although four haplotypes including TGTGCG, CGCGCG, CGTGCG, and CGTGTA were shown to have significant associations with different subphenotypes of AMD, anymore after a stringent Bonferroni correction, the significant associations no longer existed, suggesting that these haplotypes were not specifically associated with AMD. Since the haplotype CGTACA was rare in all AMD (3%) and wet groups (3%), and absent in the drusen group and controls, we think that the significant association between this haplotype and AMD is not reliable. The haplotype CGTGCA was shown to be significantly associated with soft drusen in the subject group even after a stringent Bonferroni correction (p=0.0011, Table 4). Further replication studies are needed to clarify the current situation because of the limited number of soft drusen samples in this study.
